# Phasic and Tonic mGlu7 Receptor Activity Modulates the Thalamocortical Network

**DOI:** 10.3389/fncir.2016.00031

**Published:** 2016-04-25

**Authors:** Valériane Tassin, Benoît Girard, Apolline Chotte, Pierre Fontanaud, Delphine Rigault, Mikhail Kalinichev, Julie Perroy, Francine Acher, Laurent Fagni, Federica Bertaso

**Affiliations:** ^1^CNRS, Institut de Génomique Fonctionnelle, UMR-5203Montpellier, France; ^2^INSERM, U1191Montpellier, France; ^3^UMR-5203, Université de MontpellierMontpellier, France; ^4^CNRS, UMR-8601, Université Paris DescartesParis, France; ^5^IPSEN InnovationLes Ulis, France

**Keywords:** glutamate, thalamic network, short-term plasticity, epilepsy, EEG

## Abstract

Mutation of the metabotropic glutamate receptor type 7 (mGlu7) induces absence-like epileptic seizures, but its precise role in the somatosensory thalamocortical network remains unknown. By combining electrophysiological recordings, optogenetics, and pharmacology, we dissected the contribution of the mGlu7 receptor at mouse thalamic synapses. We found that mGlu7 is functionally expressed at both glutamatergic and GABAergic synapses, where it can inhibit neurotransmission and regulate short-term plasticity. These effects depend on the PDZ-ligand of the receptor, as they are lost in mutant mice. Interestingly, the very low affinity of mGlu7 receptors for glutamate raises the question of how it can be activated, namely at GABAergic synapses and in basal conditions. Inactivation of the receptor activity with the mGlu7 negative allosteric modulator (NAM), ADX71743, enhances thalamic synaptic transmission. *In vivo* administration of the NAM induces a lethargic state with spindle and/or spike-and-wave discharges accompanied by a behavioral arrest typical of absence epileptic seizures. This provides evidence for mGlu7 receptor-mediated tonic modulation of a physiological function *in vivo* preventing synchronous and potentially pathological oscillations.

## Introduction

G protein-coupled receptors (GPCRs) represent the largest family of membrane receptors in mammals and are involved in a big diversity of patho-physiological conditions. These receptors can display constitutive (agonist-independent) activity, and mutations leading to constitutively active GPCR receptors *ex vivo* have been linked to several human diseases (de Ligt et al., 2000). However, only few reports exist on the role of constitutively active wild-type (WT) receptors in their natural environment, *in vitro* and *in vivo* (Meye et al., 2014). For instance, constitutive activity of native H3 receptors can control rat histaminergic neurons *in vivo* (Morisset et al., 2000), while constitutively active dopamine, opioid, and serotonin receptors are expressed in the brain reward system (Meye et al., 2014).

Among eight genes coding for metabotropic glutamate receptors (mGlu1-8), several studies have investigated the effects of negative allosteric modulation of WT mGlu1 and mGlu5 in neurons (Prezeau et al., 1996; Ango et al., 2001) and *in vivo* (Rodriguez et al., 2010; Keck et al., 2012), while much less is known about the constitutive activity of the other mGluRs. In the mammalian CNS, the mGlu7 receptor gene shows the highest degree of evolutionary conservation across species. It is the most widely distributed in a broad range of synapses critical for both normal CNS function as well as psychiatric and neurological disorders including anxiety, depression, obsessive–compulsive disorders, and schizophrenia (Hamilton, 2011; Shyn et al., 2011; Park et al., 2013). Different splice mGlu7 variants (a–e) exist, with mGlu7a being the predominantly expressed variant (Flor et al., 1997; Schulz et al., 2002). The mGlu7a receptor is expressed at glutamatergic presynaptic terminals (Bradley et al., 1998; Kinoshita et al., 1998), where its association to the PDZ-protein PICK1 mediates the inhibition of voltage-gated calcium channels and, as a result, a decrease in neurotransmitter release (Perroy et al., 2000, 2002; Millán et al., 2002). The activation of mGlu7 receptors remains a puzzling issue, due to its very low affinity for glutamate (in the millimolar range, amongst the lowest for neurotransmitters). Such glutamate concentration might be reached in the glutamatergic synaptic cleft upon intense neuronal activity (Cartmell and Schoepp, 2000; Pelkey et al., 2005). Intriguingly, mGlu7 receptors are also expressed at GABAergic presynaptic sites in hippocampus (Somogyi et al., 2003), hypothalamus (Schrader and Tasker, 1997), and somatosensory cortex (Dalezios et al., 2002) where the dearth of glutamate highlights the enigma of mGlu7 receptor activation and function in physiological conditions.

**FIGURE 1:**
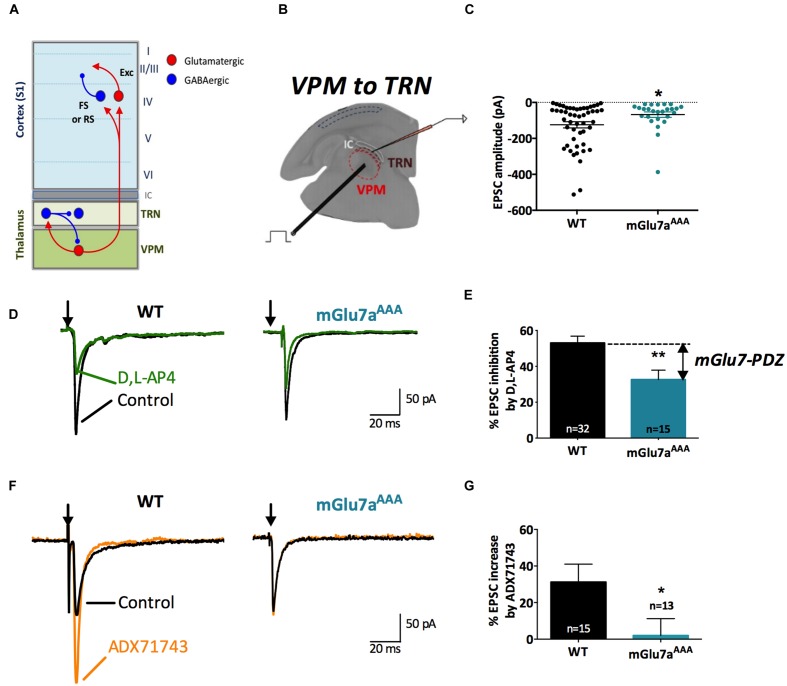
**The mGlu7 receptor modulates glutamatergic transmission at VPM synapses onto TRN neurons.** **(A)** Schematic representation of the thalamocortical and intra-thalamic connections between glutamatergic and GABAergic neurons in the somatosensory cortex (S1), the thalamic reticular nucleus (TRN) composed exclusively of GABAergic neurons, and the ventro-postero-medial nucleus (VPM), devoid of interneurons. FS, Fast-Spiking interneurons; RS, Regular-Spiking interneurons; Exc, excitatory glutamatergic neurons. **(B)** Experimental configuration, including the positions of the electrical stimulation into the VPM and the recording electrode into the TRN. **(C)** Single and average amplitudes of evoked EPSCs in WT and mGlu7a^AAA^ mice. **(D,F)** Representative evoked EPSCs recorded from TRN neurons in response to VPM stimulation of WT (left) and mGlu7a^AAA^ (right) mice and effect of D,L-AP4 (1.2 mM, **D**) and ADX71743 (10 μM, **F**). Black arrows indicate electrical stimulations. **(E)** Summary of D,L-AP4-induced inhibition of EPSCs. **(G)** Summary of ADX71743-induced increase of EPSCs. ^∗^*P* < 0.05, ^∗∗^*P* < 0.01, Mann-and-Whitney test. Error bars indicate SEM.

The mGlu7 receptor knockout mouse displays impaired fear response and higher susceptibility to alcohol and convulsants (Sansig et al., 2001; Goddyn et al., 2008). The knock-in (KI) AAA mutation of the exon coding for the mGlu7a C-terminal PDZ ligand (here named mGlu7a^AAA^ mouse) suppresses the receptor interaction with the PDZ protein PICK1. Blockade of this interaction has consequences on the receptor downstream signaling and results in loss of agonist-dependent inhibition of synaptic transmission (Perroy et al., 2002). This is sufficient to induce spatial working memory deficits (Zhang et al., 2008) and to trigger spontaneous absence-like epileptic seizures (Bertaso et al., 2008), suggesting a crucial role of mGlu7a to control neuronal network activity. We focused our attention on the thalamic circuitry responsible for absence epileptic seizures, characterized by a hypersynchronous oscillatory activity of thalamic and cortical neurons, leading to typical spike-and-wave discharges (SWD) of the thalamocortical (TC) loop in human and animal models (Crunelli and Leresche, 2002). Seizures involve reciprocal projections between two thalamic nuclei, the GABAergic reticular nucleus (TRN) and the glutamatergic ventro-postero-medial nucleus (VPM), and the somatosensory (barrel) cortex (**Figure 1A**) (Crunelli and Leresche, 2002; Steriade, 2006; Huguenard and McCormick, 2007; Beenhakker and Huguenard, 2009).

The lack of specific pharmacological tools has long been a critical impairment for the study of mGlu7 receptor function. Here, using a brain-penetrant negative allosteric modulator (NAM), ADX7174, which displays high specificity and affinity for the mGlu7 receptor (Kalinichev et al., 2013), together with a more classical orthosteric agonist (D,L-AP4) and a mouse KI mutant for mGlu7 (mGlu7a^AAA^ mouse), we revisited the function of this receptor *in vitro* and *in vivo*. We studied the functional distribution of the mGlu7a receptor in thalamic synapses in WT and mGlu7a^AAA^ mice, comparing electrically and optogenetically evoked postsynaptic responses, *in vivo* electroencephalography (EEG) and behavioral analysis. Our data provide evidence for mGlu7 tonic activity *in vivo*, which occurs at specific glutamatergic and GABAergic synapses of the thalamocortical circuitry, resulting in persistent modulation of the network activity and animal vigilance state.

## Materials and Methods

### ADX71743 Synthesis

ADX71743 was synthesized according to the following scheme (Contour et al., 2007):

**Figure:**
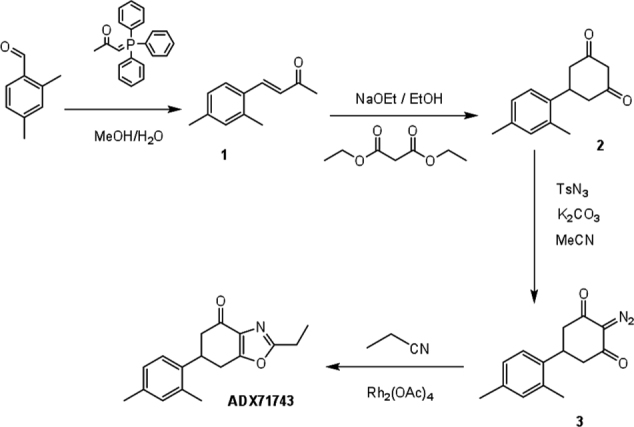


**(*E*)-4-(2,4-dimethylphenyl) but-3-en-2-one (1)** (El-Batta et al., 2007).

To a solution of 2,4-dimethylbenzaldehyde (500 mg, 3.73 mmol, 1 eq) in methanol/water 1:1 (8 ml) was added 1-triphenyl-phosphoranylidene-2-propanone (1.2 g, 3.73 mmol, 1 eq) and the mixture was refluxed for 2 h. After cooling, the solvent was removed under reduced pressure. The residue was extracted with dichloromethane (3^∗^50 ml). The combined organic layers were dried over magnesium sulfate, filtered and concentrated under vacuum. The crude was chromatographed on a silicagel column with cyclohexane/ethyl acetate (85:15) to afford **1** as a slightly yellow oil (613.6 mg, 3.53 mmol, 94.5%).

TLC: Rf = 0.47 (cyclohexane/ethyl acetate 8:2, UV).
^1^H NMR (500 MHz, CDCl_3_): δ 2.21 (s, 3H), 2.25 (s, 3H), 2.28 (s, 3H), 6.50 (d, *J* = 16.2 Hz, 1H), 6.89 (s, 1H), 6.90 (d, *J* = 8.2 Hz, 1H), 7.34 (d, *J* = 8.2 Hz, 1H), 7.67 (d, *J* = 16.2 Hz, 1H).
^13^C NMR (126 MHz, CDCl_3_): δ 19.2, 20.9, 27.2, 126.0, 126.6, 126.9, 130.1, 131.3, 137.4, 140.1, 140.2, 197.6.
MS (ESI): *m/z* 175.2 [M+H]^+^

**5-(2,4-dimethylphenyl)cyclohexane-1,3-dione (2)** (Courtney et al., 2008).

To a solution of **1** (4.36 g, 25 mmol, 1 eq) in dry ethanol (20 ml) was added NaOEt (21% in ethanol; 19.4 ml, 0.127 mol, 5 eq) and diethylmalonate (3.9 ml, 25 mmol, 1 eq) under stirring. The mixture was stirred at refluxed for 1 h 30 min. The reaction mixture was cooled to ambient temperature. An aqueous solution of sodium hydroxide (2 M, 20 ml) was added to the reaction mixture, which was heated at 80°C for 3 h. Ethanol in excess was removed by evaporation. The mixture was acidified with concentrated HCl (20 ml) and the reaction mixture was refluxed for 3 h 30′ and left to cool to ambient temperature. The compound was extracted with ether (3^∗^200 ml). The combined organic layers were dried over magnesium sulfate, filtered and concentrated under vacuum. The crude was chromatographed on a silicagel column with dichloromethane/ethyl acetate (6:4) to afford a pale yellow oil (**2**) (4.1 g, 19 mmol, 75.6%).

TLC: Rf = 0,46 (dichloromethane/ethyl acetate 85:15, UV).
^1^H NMR (250 MHz, CDCl_3_): δ 2.29 (s, 6H), 2.56–2.63 (m, 1H), 2.75–2.83 (m, 3H), 3.34–3.45 (m, 1H), 3.50–3.57 (m, 1H), 4.09–4.30 (m, 1H), 7.00–7.03 (m, 3H)
^13^C NMR (126 MHz, CDCl_3_): δ 19.5, 21.2, 37.2, 43.0, 103.4, 126.4, 127.9, 132.2, 136.3, 136.7, 140.6, 198.0.
MS (ESI): *m/z* 215.2 [M-H]^-^

**2-diazo-5-(2,4-dimethylphenyl)cyclohexane-1,3-dione (3)** (Presset et al., 2011).

To a solution of **2** (100 mg, 0.46 mmol, 1 eq) in acetonitrile (10 ml) was added a solution of *p-*toluenesulfonylazide (13% in toluene; 785 μl, 0.46 mmol, 1 eq) and K_2_CO_3_ (76 mg, 0.55 mmol, 1.2 eq). The mixture was protected from light and stirred overnight at room temperature. The mixture was then concentrated and the compound was extracted with dichloromethane (3^∗^50 ml). The combined organic layers were dried over magnesium sulfate, filtered and concentrated under vacuum. The crude was chromatographed on a silicagel column with dichloromethane/ethyl acetate (9:1) to afford a white solid (**3**) (78.2 mg, 0.32 mmol, 70.3%).

TLC: Rf = 0,76 (dichloromethane/ethyl acetate 8:2, UV and ninhydrin).
^1^H NMR (500 MHz, CDCl_3_): δ 2.27 (s, 3H), 2.28 (s, 3H), 2.63–2.74 (m, 4H), 3.53–3.58 (m, 1H), 7.00–7.04 (m, 3H).
^13^C NMR (126 MHz, CDCl_3_): δ 19.1, 20.8, 32.0, 43.7, 84.3, 124.7, 127.3, 131.8, 135.1, 136.4, 136.7, 189.5.
MS (ESI): *m/z* 243.11235 [M+H]^+^

**6-(2,4-dimethylphenyl)-2-ethyl-6,7-dihydrobenzo[*d*]oxazol-4(5*H*)-one (ADX71743)** (Kallstrom et al., 2004).

To compound **3** (2.02 g, 8.35 mmol, 1 eq) was added propionitrile (2.3 g, 41.7 mmol, 5 eq) and rhodium (II)-acetate dimer (6.88 mg, 16.7 μmol, 0.002 eq). The mixture was heated at 60°C for 1 h, cooled to room temperature and the crude was chromatographed on a silicagel column with dichloromethane/ethyl acetate (98:2) to afford a pale yellow solid (**ADX73741**) (994 mg, 3.70 mmol, 44.2%).

TLC: Rf = 0,23 (dichloromethane/ethyl acetate: 9:1, UV).
^1^H NMR (500 MHz, CDCl_3_): δ 1.41 (t, *J* = 7.6 Hz, 3H), 2.29 (s, 3H), 2.31 (s, 3H), 2.72 (dd, *J* = 16.4 and 3.9 Hz, 1H), 2.84 (d, *J* = 16.4 Hz, 1H), 2.85 (qu, *J* = 7.6 Hz, 2H), 3.10–3.13 (m, 2H), 3.83 (m, 1H), 7.06 (s, 1H), 7.07 (d, *J* = 7.8 Hz, 1H), 7.20 (d, *J* = 7.8 Hz, 1H).
^13^C NMR (126 MHz, CDCl_3_): δ 11.1, 19.4, 21.0, 21.9, 29.5, 36.9, 44.9, 125.5, 127.4, 132.0, 134.3, 135.3, 137.0, 137.1, 163.5, 166.6, 190.5
HRMS calculated for [C_17_H_20_O_2_N]^+^: 270.14940.
HRMS found for [C_17_H_20_O_2_N]^+^: 270.14862.
Elementary analysis calculated, %: C 75.81; H 7.11; N 5.20.
Elementary analysis found, %: C 75.18; H 6.95; N 4.36.
HPLC: macherey nagel (125 mm × 4.0 mm, 5 μm). Flow rate: 0.7 ml/min et λ = 254 nm.
Solvent A (water/methanol/HCOOH: 900:100:1) and solvent B (water/methanol/HCOOH 200:800:1) TR = 19.6 min.

### Mice

All animal procedures were conducted in accordance with the European Communities Council Directive (2010/63/EU) and approved by the French Ministry for Agriculture. The generation and characterization of the mutant mice are described elsewhere (mGluR7a^AAA/AAA^ mouse, herein named mGlu7a^AAA^ mice, (Zhang et al., 2008).

### Acute Thalamocortical Slices Preparation

All chemicals used to prepare electrophysiological solutions were purchased from Sigma–Aldrich (France). mGlu7^AAA^ or WT littermates male and female mice aged P15–P21 were deeply anesthetized by inhalation of isoflurane, and slices were prepared with a vibratome (Campden Instruments, UK) as previously described (350 μm-thick, 35° tilt from coronal, Cruikshank et al., 2007) to preserve the thalamocortical network. As intra-TRN projections are denser in the horizontal plan, we prepared 230 μm-thick horizontal slices when studying intra-TRN synapses. We then incubated slices at 32°C for 1 h and then at 24–26°C, in artificial cerebrospinal fluid (ACSF) containing (in mM) 120 NaCl, 2.5 KCl, 1.6 NaH_2_PO4, 1.3 MgCl_2_, 2.5 CaCl_2_, 27 NaHCO_3_, and 22 glucose, equilibrated with 95% O_2_ and 5% CO_2_, pH 7.4.

### Whole-Cell Patch-Clamp Recording

Following incubation, brain slices were transferred to the recording chamber and superfused with ACSF (2 ml/min, 32°C). For EPSC recording ACSF was supplemented with D-AP5 (50 μM) and bicuculline (10 μM). For IPSC recording, ACSF was supplemented with CNQX (10 μM) and D-AP5 (50 μM). All drugs were purchased from Tocris (France). Recordings were obtained from visually identified TRN, VPM, and cortical layer 4 neurons using differential contrast optics with an upright microscope (Olympus, France) and an infrared CCD video camera (COHU, SAIS, France). Recording electrodes made of borosilicate glass had a resistance of 4–7 MΩ when filled with intracellular solution. For thalamic neurons, the internal solution contained (in mM) 115 CsMeSO_3_, 20 CsCl, 10 HEPES, 0.6 EGTA, 4 Na_2_-ATP, 0.4 Na-GTP, 10 Na-phosphocreatine, and 2.5 MgCl_2_. For cortical layer 4 neurons, the internal solution contained (in mM) 125 K-gluconate, 10 KCl, 10 HEPES, 0.2 EGTA, 4 Na_2_-ATP, 0.3 Na-GTP, 14 Na-phosphocreatine, and 1.8 MgCl_2_. For both solution, pH was adjusted to 7.3 with CsOH or KOH, respectively, and the osmolarity was 300 mOsm. For all recording conditions, only cells with access resistance < 18 MΩ and a change of resistance < 25% over the course of the experiment were analyzed. Data were filtered with a Hum Bug (Quest Scientific, Canada), digitized at 2 kHz (Digidata 1444A, Molecular Devices, Sunnyvale, CA, USA), and acquired using Clampex 10.2 software (Molecular Devices).

### Electrical Stimulation of VPM and TRN

Electrical stimulations were delivered via a bipolar stimulation electrode (0.1–1 mA, 100 μs; FHC Inc.). To study the effect of the mGlu7 receptor at thalamic presynaptic sites, the stimulation electrode was placed either in the VPM to evoke excitatory postsynaptic currents (eEPSCs) in TRN neurons, held at -60 mV (inward currents), or in the TRN to evoke inhibitory postsynaptic currents (eIPSCs) within adjacent TRN neurons or VPM neurons, held at 0 mV (outward currents). The group III mGluRs agonist D,L-AP4 (1.2 mM, Tocris Bioscience) and the mGlu7 receptor NAM ADX71743 (10 μM) were applied and the effects compared between the two genotypes.

### Minimal Evoked Monosynaptic Responses: Single and Repetitive Stimulation

For electrical and optogenetical stimulation experiments, single-stimulation and train-stimuli were applied on slices to activate small groups of neurons within TC loop regions, and we then recorded evoked responses in neurons receiving the input. Immediately after obtaining a whole cell recording, a few test stimuli were delivered to check the synaptic responsiveness of the neuron. Once monosynaptic response was clearly observed, evoked excitatory (eEPSCs) or inhibitory postsynaptic currents (eIPSCs), respectively, were isolated by switching the perfusion solution from normal ACSF to one containing drugs (isolation of AMPA currents with ionotropic GABA and NMDA receptor blockers, 10 μM bicuculline and 50 μM D-AP5; isolation of GABA currents with ionotropic glutamate receptor blockers, 10 μM CNQX and 50 μM D-AP5; Tocris Bioscience). After 7 min drugs perfusion, test stimuli of increasing intensity were applied to determine the minimal excitability threshold evoking monosynaptic postsynaptic currents. Stimulation vs. amplitude values for all cell type tested are reported in **Supplementary Figure 1**. Averages of 10–20 trials were used to measure the amplitudes of ePSCs, from baseline to peak.

We also tested the response plasticity by applying five pulses (0.1–1 mA, 100 μs duration) at 10 Hz, close to natural whisker-input frequency into the thalamus during exploratory whisking (Knutsen et al., 2008). Averaged 10 trials of 10 s inter-trials were used to measure the five peak amplitudes. Short-term plasticity depends on a presynaptic modulation of neurotransmitter release, which is reflected postsynaptically by an increase (synaptic facilitation) or decrease (synaptic depression) of ePSCs along the stimulation train. Facilitation and depression ratios were calculated as the percentage of the response amplitude to pulse 2 to 5 divided by the response amplitude to the pulse 1. In experiments in which we added supplementary drugs in the perfusing solution, we repeated single-stimulation and train-stimuli protocols after 7 min.

### Spontaneous Phasic and Tonic GABA_A_ Currents Recordings

Thalamic reticular nucleus and VPM cells receiving TRN neurons inputs were voltage-clamped at -60 mV, and GABA_A_ receptor-mediated currents were pharmacologically isolated with 10 μM CNQX and 50 μM D-AP5. Miniature IPSCs (mIPSCs) were obtained by adding 1 μM tetrodotoxin (TTX, Tocris) to block action potentials. mIPSCs frequency was analyzed and compared between WT and mGlu7a^AAA^ mice. Tonic GABA_A_ currents were revealed as a shift in baseline current following the addition of the GABA_A_ receptor antagonist gabazine (SR-95531, Sigma–Aldrich) to the perfusing solution.

### Optogenetical Stimulation of Thalamocortical Fibers

Adeno-associated virus carrying cDNA for channelrhodopsin and the fluorescent reporter mCherry (AAV2/1.CAG.hChR2(H134R)-mCherry; Karl Deisseroth, Stanford University via the UPenn VectorCore, PA, USA) were injected into the VPM of WT or mGlu7^AAA^ mice at post-natal days 13 (stereotaxic coordinates from olfactive bulb junction were lateral, 2.10 mm; posterior, 4.50 mm; and depth, 3.50 mm). The viral titer was 10^11^ IU/ml and injection volume was 0.7 μl. After allowing 4–8 days for ChR2 expression, acute somatosensory thalamocortical slices were used for *in vitro* patch-clamp recording (see above). VPM synaptic terminals expressing hChR2-mCherry were stimulated with blue light (LED, 488 nm, 0.1 ms/pulse, 40–200 μW) shined in the barrel cortex layer IV. Cortical neurons were identified as excitatory cells, Regular-Spiking (RS) or Fast-Spiking (FS) interneurons, according to their electrophysiological properties (see **Supplementary Figure 2**). In some cases, at the end of the experiments TC slices were incubated overnight in phosphate buffer saline solution (PBS) containing 4% PFA at 4°C and mounted between microscope slides and coverslips using Mowiol to image the correct localization of virus-induced expression of hChR2-mCherry with an AxioImager Z1 apotome and AxioVision software (Carl Zeiss, France).

**FIGURE 2:**
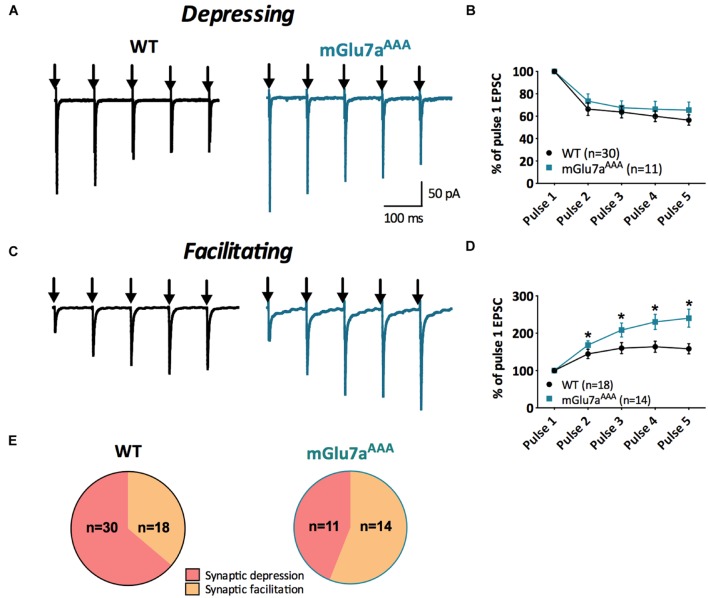
**Short-term synaptic plasticity in VPM to TRN synapses.** **(A,C)** Representative current traces from TRN neurons responding to five stimuli at 10 Hz (black arrows) in WT (left) and mGlu7^AAA^ (right) mice. VPM afferents display either short-term synaptic depression **(A)** or facilitation **(C)** in both genotypes. **(B,D)** Short-term synaptic depression ratios show no significant differences between the two genotypes, while facilitation ratios are strongly increased in mGlu7^AAA^ mice. **(E)** Proportion of neurons displaying either short-term synaptic depression or facilitation in WT (left) or mGlu7a^AAA^ (right) mice. ^∗^*P* < 0.05, Mann-and-Whitney test. Error bars indicate SEM.

### Surgery and Electroencephalography (EEG) Recordings

Wild-type or mGlu7^AAA^ male mice were anesthetized with 2 ml/kg of a saline solution containing 40% (v/v) ketamine (Imalgene 500) and 20% (v/v) xylazine (Rompun 2%) and placed in a stereotaxic frame using the David Kopf mouse adaptor. A male microconnector (Pinnacle Technology Inc., Lawrence, KS, USA) was fixed by four extradural screws, two on each parietal bone. The microconnector was then fixed with dental acrylic cement. After surgery, animals were individually housed and maintained in a 12 h light/dark cycle with food and water *ad libitum* for 1 week of recovery.

For recordings, animals were put into individual Plexiglas boxes, and their micro connectors were plugged to an EEG preamplifier circuit and to the EEG amplifier (Pinnacle Technology Inc., Lawrence, KS, USA). The electrical activity recorded by extradural electrodes was filtered at 50 Hz, sampled at 400 Hz and recorded by a computer equipped with Sirenia^®^ software (Pinnacle Technology Inc.). A 30 min period of baseline recording before ADX71743 injection (i.p.; volume of injection: 100 μl/10 g mouse weight of a 100 mg/kg suspension in 50% v/v H_2_0 and hydroxyl-propyl-β-cyclodextrin) allowed detection of EEG abnormalities independent of experimental manipulations. Control animals were injected with vehicle alone. EEG recordings were performed together with monitoring animal behavior. The symptoms developing after injection were classified into different phases as described by (Weiergraber et al., 2006)): phase 0 corresponds to normal exploration and posture; phase 1 corresponds to an “absence-like” non-convulsive state, with reduced motility and typical prostrated position of the animal; phase 2 shows partial clonus of head, vibrissae and/or forelimbs; phase 3 consists of a generalized clonus of the extremities, the tail, and sometimes vocalization; this phase can develop into typical “running fits”; phase 4 corresponds to a generalized tonic-clonic seizure with extension of the four paws; this phase is occasionally followed by death by respiratory failure. We defined “lethargy” as a continuous period of immobility or slow movement of the mouse limited to less than one body-length of the mouse. Observations were made by a blind observer.

### HEK293 Transfection

In order to enhance the read-out of our pharmacological tests, we used the chimeric Gq/io protein that is able to bind all GPCRs (GqTOP). Stimulation of the mGlu7/GqTOP pathway activates phospholipase C (PLC), which hydrolyzes phosphatidylinositol bisphosphate (PIP2) to form two second messengers, inositol 1,4,5-triphosphate (IP3) and diacylglycerol (DAG). IP3 is very rapidly hydrolyzed to IP2 then to IP1, and finally to inositol by a series of enzymatic reactions (Berridge and Taylor, 1988) blocked by lithium chloride (LiCl; Garbison et al., 2012). HEK293 cells were transfected by electroporation (250 V–500 μF for 5.10^6^ cells) with plasmids expressing the GqTOP protein and the glutamate transporter EAAC1, associated with vehicle plasmids (control, MOCK) or mGlu7a-expressing plasmid (Perroy et al., 2000). Cells were plated at 150,000 per well (each condition in triplicate) in 96-well sterile, black microplates in DMEM supplemented with 10% FCS. After 24 h, DMEM medium was replaced by Glutamax^TM^ (GIBCO, Thermo Fisher, France) 2 h before performing TR-FRET experiments. Notice that the expression of EAAC1 as well as Glutamax^TM^ allowed obtaining a minimal glutamate extracellular concentration, to avoid glutamate-mediated activation of mGlu7a receptors.

### Inositol Phosphate 1 Measurement by Time-Resolved Fluorescence Resonance Energy Transfer (TR-FRET) Assay

The production of IP1 by HEK293 cells was tested using TR-FRET assays, based on Time-Resolved Resonance Energy Transfer. TR-FRET assays were performed in presence of LiCl to block IP1 degradation, using anti-IP1 mouse antibody labeled with Terbium cryptate-conjugate (Tb, Tb-Ab) that will be the donor of energy, and d2-conjugated IP1 (d2, d2-IP1) that will be the acceptor of energy (Cisbio Bioassays, France). After 30 min incubation at 37°C with stimulation buffer alone (≪ Base ≫) or supplemented with a group III mGluRs agonist (L-AP4, 600 μM) or antagonist (CPPG, 100 μM, Tocris Bioscience, France) or the mGlu7-specific NAM ADX71743 (10 μM), d2-IP1 acceptor and Tb-Ab donor were added and cells incubated for 1 h at room temperature. The anti-IP1 antibody competes with native IP1 produced by cells and D2-labeled IP1 from the kit. The fluorescence of Tb and d2 were measured, respectively, at 620 and 665 nm (after 50 μs delay) and divided by the 337 nm excitation signal using a RUBYstar plate reader (BMG, Germany). The resulting signal is inversely proportional to the concentration of IP1 in samples, determined by using a standard curve construction, since the donor–acceptor pair gives a high transfer of energy signal that decreases upon competition with IP1 produced by the cells (Garbison et al., 2012).

### Data Analysis

Whole-cell recordings were analyzed using Clampfit 10.2 software (Molecular Devices). Response amplitudes, short-term plasticity ratios, mIPSCs properties and tonic current amplitudes were compared between WT and mGlu7a^AAA^ mice using the Mann-Whitney test or repeated measures ANOVA (^∗^*P* < 0.05, ^∗∗^*P* < 0.01, ^∗∗∗^*P* < 0.001). Normalized IP1 concentrations obtained from TR-FRET experiments were compared using an unpaired *t*-student test (^∗^*P* < 0.05, ^∗∗^*P* < 0.01, ^∗∗∗^*P* < 0.001). All averaged data are presented as mean ± SEM.

Eelectroencephalography signals were analyzed using Matlab 2013b software (Matworks Inc.). Short time Fourier transform analysis was realized using the Signal Processing toolbox (Spectrogram function) and applied to the EEG data in order to obtain the energy of the signal as a function of frequency and time (Freeman and Quiroga, 2013). Bartlett-type window was used to avoid leakage.

We extracted a qualitative measure for each of the EEG frequency bands i (i = δ, 𝜃, α, β, γ), where δ = 2 – 4 Hz, 𝜃 = 4 – 8 Hz, α = 8 – 14 Hz, β = 14 – 30 Hz, and γ = 30 – 70. This measurement is defined as:

$$I^{i}(t)=\int_{f_{\min}^{i}}^{f_{\max}^{i}}I(f,t)df\iota =\delta ,\theta ,\alpha ,\beta ,\gamma \,$$

where (f_min_^i^,f_max_^i^) are the frequency limits for the band *i*, and *I(f,t)* is the power spectrum as a function of frequency *f* and time *t*. The time windows used 256 points (0.64 s at a sampling rate of 400 Hz).

## Results

### Modulation of Glutamatergic Thalamic Synapses by mGlu7a Receptor PDZ-Dependent Signaling

We aimed at identifying the thalamic synapses functionally expressing mGlu7a receptors, by comparing the response of WT mice to mGlu7a^AAA^ mice, which are deficient in controlling neurotransmitter release (Zhang et al., 2008).

Intra-thalamic transmission properties were first tested by electrically stimulating VPM neurons and recording evoked excitatory postsynaptic currents (EPSCs) in the TRN on *in vitro* TC slices (**Figure 1B**). Neurons from mGlu7a^AAA^ mice showed smaller EPSCs than WT neurons (WT: -124.3 ± 17.9 pA, *n* = 48; mGlu7a^AAA^: -68.0 ± 15.2 pA, *n* = 28, *p* = 0.050; **Figure 1C**). This observation was not due to a change in input resistance, which was similar in WT and mGlu7a^AAA^ TRN neurons (261 ± 90 MOhm for WT, 272 ± 43 MOhm for mGlu7a^AAA^, *p* = 0.9088). The non-selective mGlu7 receptor agonist, D,L-AP4 (1.2 mM), induced the inhibition of evoked EPSC. This was significantly smaller in mGlu7a^AAA^ (32.6 ± 5.3%, *n* = 15) than in WT neurons (53.0 ± 3.8%, *n* = 32; *p* = 0.0034; **Figures 1D,E**). The difference between the two values represents the mGlu7a-PDZ ligand-dependent pathway activation. The residual inhibition induced by D,L-AP4 likely resulted from action of the drug on mGlu4 and/or mGlu8 receptors, or PDZ ligand-independent mGlu7 receptor signaling. This applies to all the synapses we will analyze in the following experiments. Analysis of amplitude response as a function of stimulation intensity is given in **Supplementary Figure 1**. For neurons requiring low stimulation intensity, the responses of WT and mGlu7a^AAA^ synapses were similar, suggesting that the properties of postsynaptic receptors are not involved. However, for neurons requiring higher stimulation intensities, which require more available vesicles, WT synapses respond more strongly than mGlu7a^AAA^ synapses, suggesting that the pool of vesicles in the mGlu7a^AAA^ neurons is limiting. This is in keeping with a greater proportion of neurons presenting a facilitating response in mGlu7a^AAA^ VPM to TRN synapses, as smaller initial responses have a greater potential to facilitate (see for example Zucker and Regehr, 2002). Another possibility is that a lower number of fibers is recruited upon stimulation in mGlu7a^AAA^ mice as a consequence of developmental modifications of network excitability.

We also found that eEPSCs were increased by the negative allosteric mGlu7 modulator (NAM) ADX71743 (10 μM) in WT mice (31.2 ± 9.8%; *n* = 15), highlighting a tonic activity of the receptor. The effect of the NAM was much smaller in mGlu7a^AAA^ mice (1.9 ± 9.2%; *n* = 13; *p* = 0.0111; **Figures 1F,G**). These results suggest the existence of an mGlu7a receptor PDZ-dependent inhibition of glutamatergic VPM projections within the TRN in WT mice.

As previously described (Mistry et al., 2008), a 10 Hz 5-pulse stimulation protocol applied to VPM-TRN synapses induced either synaptic depression or synaptic facilitation, in both WT and mGlu7a^AAA^ mice (**Figure 2**). Depression ratios were non-significantly modified in mutant mice (**Figures 2A,B**), while facilitation ratios were significantly higher compared to WT mice (**Figures 2C,D**). Moreover, the proportion of neurons showing a facilitating response was significantly increased in mGlu7a^AAA^ mice (**Figure 2E**). These data indicate that an active mGlu7a receptor PDZ-dependent pathway promotes the low-pass filtering of information from the VPM to the TRN by increasing synaptic depression and lessening facilitation in basal conditions. Interestingly, treatment with ADX7173 did not change the short-term plasticity (data not shown), likely a consequence of a long-term absence of mGlu7a C-terminal signaling. The lower EPSCs amplitudes in mGlu7a^AAA^ mice seen in **Figure 1C** might reflect presynaptic fatigue due to the over-excitation of synaptic terminals. The tonic activity of mGlu7a receptors might lead to an inhibition of voltage-gated calcium channels, which would decrease Ca^2+^ entry into WT synapse terminals. Another possibility would be that postsynaptic glutamate receptors desensitize upon sustained activation. This would be consistent with our results showing that the synaptic facilitation is favored and depression is reduced in mutant mice, in the absence of exogenous agonists.

### Modulation of GABAergic Synaptic Transmission within and from Thalamic Reticular Neurons by mGlu7 Receptors

**FIGURE 3:**
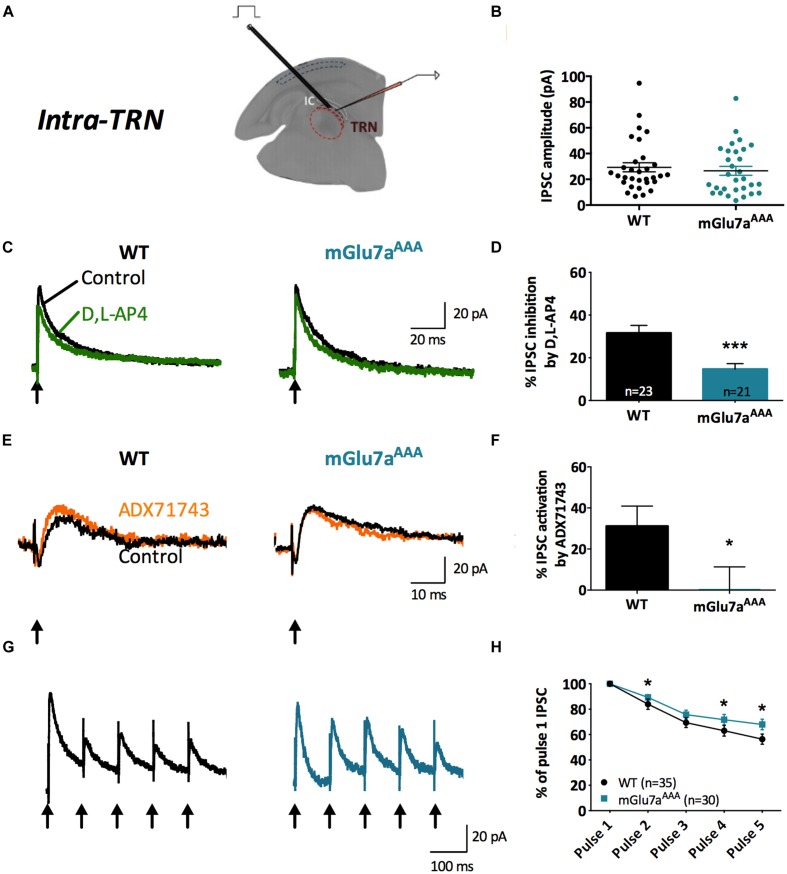
**The mGlu7 receptor modulates GABAergic transmission and short-term synaptic plasticity in intra-TRN synapses.** **(A)** Experimental configuration, including positions of electrical stimulation into the TRN and recording in adjacent reticular neurons. **(B)** Evoked IPSC amplitudes show no difference in responsiveness between WT and mGlu7^AAA^ mice. **(C,E)** Representative IPSC recorded from TRN neurons in response to TRN single stimulation in WT (left) and mGlu7a^AAA^ (right) mice before and after application of D,L-AP4 **(C)** or ADX71743 **(E)**. Black arrows indicate electrical stimulations. **(D)** D,L-AP4-induced IPSC inhibition. **(F)** ADX71743-induced EPSC increase. **(G)** Representative traces in response to 5 × 10 Hz stimuli and **(H)** short-term synaptic depression ratios. ^∗^*P* < 0.05, ^∗∗∗^*P* < 0.001, Mann-and-Whitney test. Error bars indicate SEM.

**FIGURE 4:**
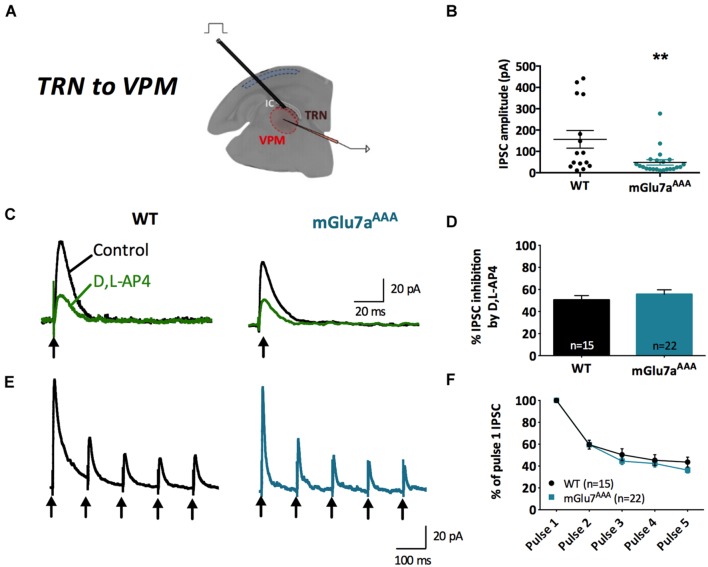
**The mGlu7 receptors modulate TRN neurons-mediated tonic GABA_A_ inhibition in the VPM.** **(A)** Experimental configuration, including positions of recording electrode into the VPM and electrical stimulus on TRN. **(B)** Single and average IPSC amplitudes in WT and mGlu7a^AAA^ mice. **(C,E)** Representative IPSC traces from VPM neurons responding to TRN single **(C)** or 5 × 10 Hz **(E)** stimulation of WT (left) and mGlu7a^AAA^ (right) mice. Black arrows indicate electrical stimulations. **(D,F)** D,L-AP4-induced IPSC inhibition and short-term synaptic depression ratios are similar in both genotypes. ^∗∗^*P* < 0.01, Mann-and-Whitney test. Error bars indicate SEM.

Thalamic reticular nucleus neurons innervate adjacent reticular neurons as well as VPM neurons and constitute the only inhibitory input in these two nuclei in mice (Pinault and Deschenes, 1998). We investigated the synaptic function of the mGlu7 receptor on synaptic transmission and short-term plasticity by electrically stimulating TRN neurons, and recording the evoked IPSCs into either TRN or VPM nuclei in TC slices (**Figures 3** and **4**).

Amplitudes of intra-TRN IPSCs evoked by single stimulations were similar in WT and mGlu7a^AAA^ slices (**Figure 3B**). D,L-AP4 inhibited intra-TRN evoked IPSCs and this effect was reduced in mGlu7a^AAA^ mice (14.7 ± 2.6%, *n* = 21) as compared to WT mice (31.6 ± 3.6%, *n* = 23; *p* < 0.001; **Figures 3C,D**). Interestingly, we found that the amplitudes of eIPSCs were increased by ADX71743 in WT mice (31.2 ± 9.7%; *n* = 12). In this context the contribution of endogenous glutamate release to activation of the receptor should be limited, suggesting that the mGlu7 receptor constitutively inhibits GABA release by TRN neurons. Conversely, the effect of the NAM was minimal in mGlu7^AAA^ mice (0.56 ± 10.8%; *n* = 13; *p* = 0.0297; **Figures 3E,F**), implying that this constitutive activity is dependent on mGlu7 PDZ ligand-binding site.

In accordance with previous studies (Reichova and Sherman, 2004), we found that repetitive stimulation induced depression of intra-TRN evoked IPSCs. This effect was significantly reduced in mGlu7a^AAA^ mice (**Figures 3G,H**), thus reflecting a reduced plasticity of GABA synapses.

### mGlu7a Receptors Reduce Tonic GABA Currents in the VPM

At TRN–VPM synapses (**Figure 4A**), we found that phasic, D,L-AP4-induced inhibition as well as synaptic depression were similar in WT and mGlu7a^AAA^ mice (**Figures 4C,D**), implying a non-contribution of mGlu7a receptor PDZ-dependent pathway in the control of those synapses. However, we found that eIPSCs were smaller in mGlu7a^AAA^ mice (**Figure 4B**), a phenomenon that might be due to indirect fatigue following overactivation of the intra-TRN pathway.

In synthesis, the PDZ-dependent pathway of mGlu7a receptor modulates evoked inhibitory synaptic transmission only in intra-TRN, but not in TRN–VPM synapses.

**FIGURE 5:**
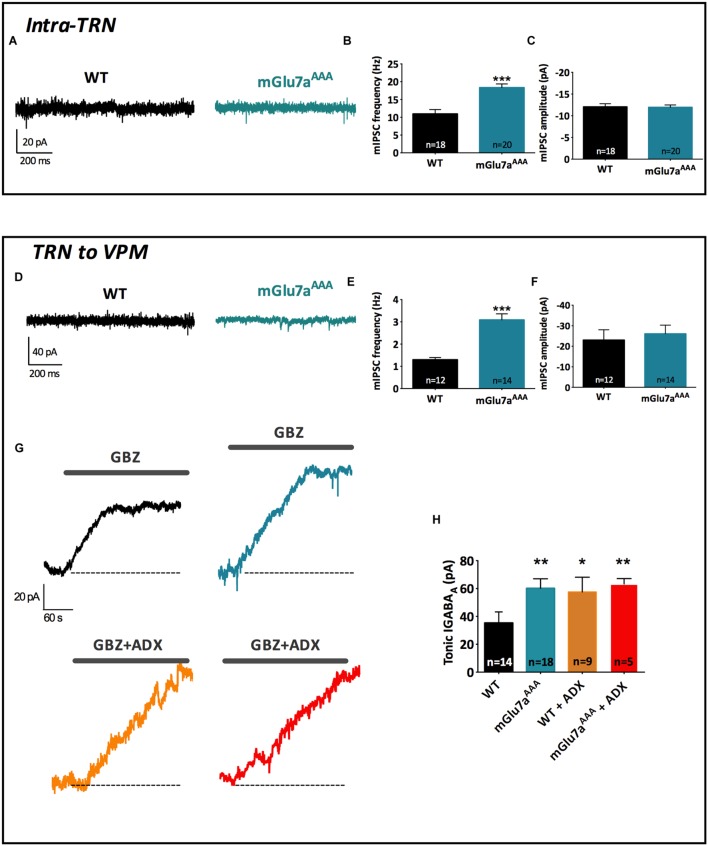
**The mGlu7a receptor decreases TRN neurons-mediated tonic and phasic GABA_A_ inhibition in VPM and TRN.** **(A)** Representative current traces of spontaneous mIPSCs recorded from WT and mGlu7^AAA^ TRN neurons in the presence of TTX (1 μM). **(B,C)** Mean mIPSCs frequency **(B)** and amplitude **(C)** in TRN neurons. **(D,E,F)** Same legend as in **(A–C)**, respectively, in VPM neurons. **(G)** Example traces of tonic GABA_A_ current recorded in WT and mGlu7^AAA^ TRN neurons upon application of 50 μM gabazine (top traces, black and blue) or gabazine plus ADX71743 (10 μM, bottom traces, orange and red). The baseline shift reflects the inhibition of tonic GABA_A_ current. **(H)** Average tonic GABA_A_ current amplitude in WT and mGlu7^AAA^ neurons with and without co-application of ADX71743 (10 μM). ^∗^*P* < 0.05, ^∗∗^*P* < 0.01, ^∗∗∗^*P* < 0.001, Mann-and-Whitney test. Error bars indicate SEM.

The above results showed that mGlu7a receptors control evoked synaptic activity in the thalamus. Here, we investigated whether the mGlu7a receptor could also affect the spontaneous synaptic activity of reticular neurons. We first examined phasic GABA_A_ inhibition in neurons from WT and mGlu7^AAA^ TC slices. mIPSC were studied in both the TRN and VPM neurons, which receive GABAergic inputs exclusively from TRN neurons. The frequency of mIPSCs was higher in both TRN (WT: 1.30 ± 0.10 Hz, *n* = 12; mGlu7^AAA^: 3.09 ± 0.27 Hz, *n* = 14; *p* < 0.0001; **Figures 5A,B**) and VPM neurons (WT: 10.92 ± 1.23, *n* = 18; mGlu7^AAA^: 18.34 ± 1.05, *n* = 20; *p* < 0.0001; **Figures 5D,E**) of mGlu7^AAA^ than in WT mice, while mIPSCs amplitudes were identical in both genotypes (**Figures 5C,F**). Enhanced mIPSCs frequency could be due to long-term change of the release machinery and/or differential presynaptic bouton handling of calcium levels.

Altogether these results suggest the implication of the mGlu7a receptor PDZ-dependent pathway in spontaneous GABA release from TRN neurons.

Synaptically released GABA can also diffuse outside the synapse and activate extra-synaptic GABA_A_ receptors, leading to a tonic GABA_A_ current (Mody, 2001) that can be specifically blocked by gabazine (Cope et al., 2005). Using this pharmacological tool, we found that tonic GABA_A_ current amplitude was increased in the VPM of mGluR7a^AAA^ slices (WT: 35.23 ± 7.96, *n* = 14; mGlu7a^AAA^: 60.20 ± 6.83, *n* = 18; *p* < 0.01; **Figures 5G,H**), suggesting that extra-synaptic GABA level in the VPM is higher in mGlu7^AAA^ than in control mice and reinforcing the idea of an essential basal modulation of TRN activity by the mGlu7 receptor. Indeed, application of ADX71743 (10 μM) on WT VPM neurons increased the gabazine-sensitive current to levels comparable to those found in mGlu7a^AAA^ mice and had no effect on mGlu7a^AAA^ mice (**Figure 5H**). These results suggest that mGlu7 receptors tonically inhibit GABA release on VPM neurons. Conversely, tonic GABA_A_ current was absent in TRN neurons of both WT and mGlu7a^AAA^ mice (data not shown), which is in accordance with the absence of extra-synaptic GABA_A_ receptors in this nucleus (Jia et al., 2005).

### mGlu7 Receptors Depress VPM to Cortical Layer 4 Fast-spiking Interneuron Synapses

Thalamic neurons relay sensory information to the barrel cortex, and VPM axon terminals project predominantly onto cortical layer 4, where three main classes of neurons are found: glutamatergic neurons (i.e., pyramidal and spiny stellate neurons), and the RS and FS GABAergic interneurons. They are so-called on the basis of their firing pattern in response to current injection, as confirmed by our characterization (**Supplementary Figure 2**).

**FIGURE 6:**
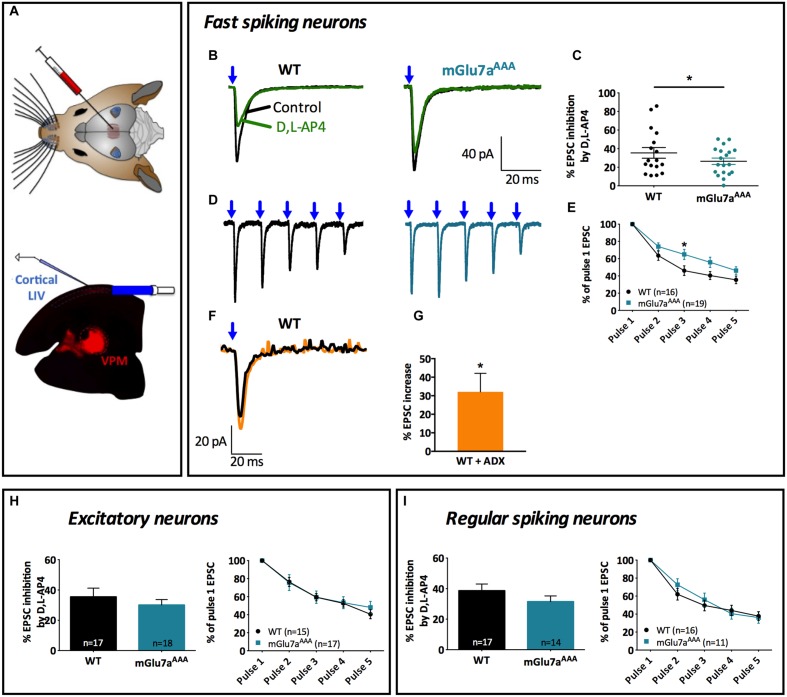
**The mGlu7 receptor modulates glutamatergic transmission between VPM neurons and cortical layer 4 Fast-Spiking interneurons.** **(A)** Experimental configuration and example of a thalamocortical slice expressing hChR2-mCherry in VPM thalamic neurons and their axons targeting cortical layer 4. LED stimuli (blue light, 488 nm) were directed on layer 4 thalamocortical hChR2-expressing terminals where postsynaptic responses of cortical neurons were recorded. **(B,D)** Representative current traces recorded from a Fast-Spiking interneuron in response to VPM single **(B)** or 5 × 10 Hz **(D)** stimulations, in WT (left) and mGlu7a^AAA^ (right) mice. Blue arrows indicate light stimulations. **(C)** D,L-AP4-induced EPSC amplitude inhibition (*n* = 17 for WT, *n* = 19 for mGlu7a^AAA^ mice). **(E)** Synaptic depression ratios in WT (left) and mGlu7a^AAA^ mice. Please note the decrease in synaptic depression in the mGlu7^AAA^ mice. **(F)** Representative current traces showing the increase in EPSC by ADX71743. **(G)** Summary of the effects of ADX71743 (10 μM) on EPSCs. **(H,I)** Average EPSC inhibition induced by D,L-AP4 and short-term synaptic depression ratios in excitatory neurons and Regular-Spiking interneurons, respectively. Please note the absence of difference between WT and mGlu7a^AAA^ mice. ^∗^*P* < 0.05, Mann-and-Whitney test. Error bars indicate SEM.

As thalamo-cortical and cortico-thalamic fibers follow the same path, we used an optogenetic approach to selectively stimulate thalamocortical afferents in brain slices (Cruikshank et al., 2010). Channelrhodopsin 2 (hChR2) was expressed in VPM neurons by AAV-mediated viral infection and eEPSCs were induced in cortical layer 4 neurons by shining blue light (488 nm) in this area (**Figure 6A**).

The inhibition of evoked EPSCs by D,L-AP4 in FS interneurons was smaller in mutant mGlu7a^AAA^ than in WT mice (26.4 ± 3.4% vs. 39.0 ± 3.5%; *p* = 0.023; **Figures 6B,C**). VPM-FS synapses showed depression upon repetitive stimulation (**Figures 6D,E**), as described in previous studies (Cruikshank et al., 2010), but depression ratios were significantly smaller in mGlu7a^AAA^ than in control mice FS interneurons. These data suggest a regulation of cortical layer 4 FS interneuron activity by VPM afferents involving an mGlu7a receptor PDZ-dependent pathway.

We then investigated the effects of negative allosteric modulation of the mGlu7 receptor-mediated inhibition of FS interneurons EPSCs in TC slices from WT mice. ADX71743 increased FS interneuron EPSC amplitude evoked by single stimulation of VPM axons (**Figures 6F,G**).

On the contrary, inhibition of single evoked EPSCs as well as synaptic depression ratio upon repetitive VPM afferent stimulations (**Figures 6H,I**) recorded from cortical layer 4 glutamatergic neurons and RS interneurons were similar in both mice genotypes. Taken together, these results indicate an mGlu7a receptor PDZ dependent-pathway mediated inhibition of VPM glutamatergic contacts innervating cortical layer 4 FS interneurons, but not other studied cortical cell types. They also suggest that mGlu7 receptors tonically inhibit excitation of FS interneurons by VPM afferents.

### Potential Constitutive Activity of mGlu7 Receptors

**FIGURE 7:**
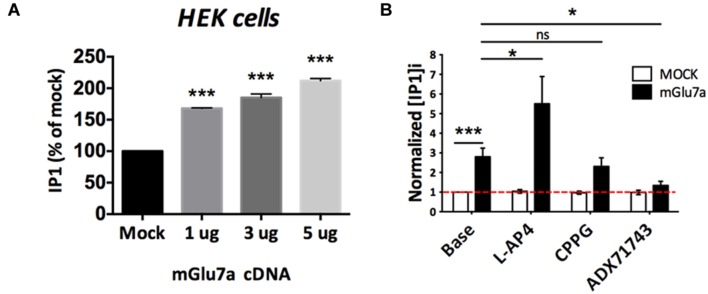
**Constitutive activity of mGlu7a receptors in the absence of extracellular glutamate.** **(A)** Normalized IP1 production in HEK cells transfected or not (Mock) with increasing amount of mGlu7a receptor cDNA (*n* = 3). Note that transfection of mGlu7a receptor in the absence of agonist induces IP1 synthesis. **(B)** IP1 formation measured in the absence (Base) and presence of the indicated compounds in HEK cells transfected with either wild-type (WT) or a control empty vector (*n* = 5). ^∗^*P* < 0.05, ^∗∗∗^*P* < 0.001, Mann-and-Whitney test. Error bars indicate SEM.

The lack of mGlu7-specific orthosteric antagonist prevented us to definitively conclude on whether mGlu7 has a constitutive, agonist-independent activity in acute TC slices. Nevertheless we investigated whether the mGlu7 receptor NAM ADX71743 displayed a true inverse agonist activity in the virtual absence of glutamate. Inverse agonists are a class of pharmacological compounds that block receptor constitutive activity. We transfected HEK cells with different amounts of mGlu7a receptor cDNA and measured receptor activity as inositol production by using a chimeric G protein-based time-resolved FRET assay (TR-FRET, see Section “Materials and Methods” for details). Importantly, co-transfection of the excitatory amino-acid transporter EAAC1 allowed lowering the extracellular glutamate concentration to a sub-micromolar range, unable to activate mGlu7a receptors (P. Rondard, IGF Montpellier, personal communication). We remarked a higher receptor activity as compared to the control transfection, which was increasingly proportional to the quantity of mGlu7a receptor expressed (**Figure 7A**). This suggested a constitutive activity of the recombinant receptor. We then compared the effect of the orthosteric non-selective mGlu7a receptor agonist L-AP4 (600 μM), and antagonist CPPG (100 μM), as well as the selective mGlu7 receptor NAM ADX71743 (10 μM) on the production of inositol phosphates (IP) in mGlu7a receptor-transfected HEK cells (**Figure 7B**). In mGlu7a-transfected cells, IP1 production increased in the presence of L-AP4, confirming that the recombinant receptor was functionally expressed in HEK cells. The basal production of IP1 in HEK cells expressing mGlu7a receptors was not modified by CPPG, as expected from a classical orthosteric antagonist in the absence of agonist. On the contrary, ADX71743 inhibited basal IP1 production in the absence of agonist, likely resulting from constitutive activity of the recombinant mGlu7a receptor.

### *In Vivo* Induction of Absence Epileptic Seizures and Lethargy by the mGlu7 Receptor Inverse Agonist

**FIGURE 8:**
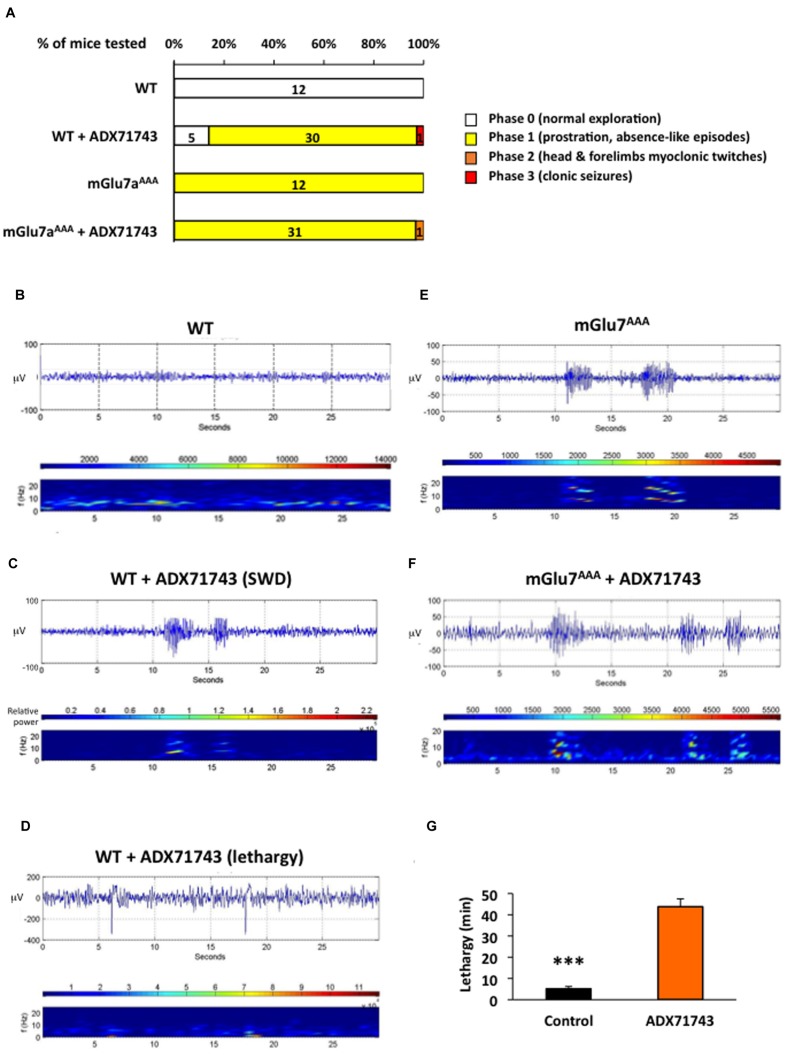
**EEG activity induced by treatment with the mGlu7 receptor NAM.** **(A)** Behavioral response of mice WT and mGlu7^AAA^ mice treated with the either vehicle or mGlu7 NAM (100 mg/kg i.p.) represented as percentage of total animals tested (n for each score group is indicated on the histogram bars). The scoring scale is given on the right. **(B–F)** Example of EEG traces and corresponding short-time Fourier transform representation (frequency over time) are shown for WT mice injected with either the vehicle alone **(B)** or the mGlu7 NAM **(C,D)** and for representative mGlu7^AAA^ mice showing spontaneous SWD when treated with the vehicle **(E)** or with the mGlu7 NAM **(F)**. Traces in **(C,D)** show, respectively, SWD-like and low frequency activity with recurrent spikes corresponding to K-complexes. The level of wavelet power is represented using the color scale. **(G)** Latency and duration of the lethargic state observed in WT mice upon ADX71743 administration.

What is the physiological role of the mGlu7 receptor tonic activity? We examined this issue *in vivo* by comparing the effects of ADX71743 (100 mg/kg i.p.) or vehicle alone (β-cyclodextrin, CD) on brain activity and behavior in WT and mGlu7a^AAA^ mice, where neurotransmitter release inhibition mediated by mGlu7a receptor is lost. CD-injected (control) mice continued normal exploratory activity and grooming, and showed no modification of their EEG pattern. The majority of WT mice (31 out of 36 mice) injected with ADX71743 showed a net decrease in exploratory activity and reactivity, entering a phase of prostration (**Figure 8A**). Such a lethargic status appeared 5 ± 0.5 min after injection and lasted 43.8 ± 3.7 min (**Figure 8G**). The mGlu7a^AAA^ mutant mice showed the typical absence-like behavior with repeated episodes of exploratory activity arrest and facial myoclonus previously reported (Bertaso et al., 2008). Administration of ADX71743 did not worsen this phenotype (31 out of 32, **Figure 8A**).

The analysis of EEG in WT mice treated with the ADX71743 revealed an increase in the power of the 0–4 Hz delta band and 4–8 theta band (Niedermeyer and Lopes Da Silva, 1993), with appearance of slow-wave spindles (2–8 Hz) with recurrent spikes typical of K-complexes of non-REM sleep compared to control mice (**Figures 8B,D**, *n* = 21 mice). In four cases out of 21, on top of the described sleep-related activity, we observed SWD with an intrinsic frequency of 8–10 Hz (**Figure 8C**), similar to those spontaneously arising in non-treated mGlu7a^AAA^ mice (**Figure 8E** and Bertaso et al., 2008). Administration of ADX71743 to mGlu7a^AAA^ mice did not increase the shape, number or duration of SWD (**Figure 8F**). More details on frequency band analysis can be found in **Supplementary Figure 3**. These data show that pharmacological blockade of mGlu7 receptor tonic activity *in vivo* induces a lethargic state accompanied by abnormal sleep-related behavior and absence epileptic seizures.

## Discussion

Despite its wide distribution and conservation among species (Flor et al., 1997; Kinoshita et al., 1998), the function of mGlu7 receptor in the brain remains poorly understood. The homology with the other mGluRs has to date hindered the design of type-specific orthosteric agonists and antagonists. Here, we took advantage of the combination of a genetic mouse model and a new inverse agonist to challenge the classical view of mGlu7 as a receptor exclusively activated when glutamate concentration rises high. Our results show that mGlu7 receptors are functionally expressed at synapses formed onto and between TRN neurons, as well as VPM synapses onto fast-spiking interneurons of the somatosensory cortical layer 4. We uncover the modulation of the tonic GABA current by mGlu7 receptors in TRN synapses. Furthermore, we demonstrate the involvement of the mGlu7a receptor PDZ-dependent pathway in short-term plasticity enhancement, reinforcing depression, and reducing facilitation in WT mice. Mutation of the C-terminal PDZ ligand motif changes the balance between facilitation and depression in synapses onto TRN neurons and cortical FS interneurons in mGlu7a^AAA^ mice, an effect that might result from the long-term absence of mGlu7 in the TC network. Previous work has shown that expression of the closely related mGlu4 receptor is not changed in the mGlu7a^AAA^ mouse, nor is the localization of mGlu7 (Zhang et al., 2008). However, the results were not specific to the thalamocortical circuit. Also, the possibility that other proteins involved in the mGlu7/PICK1 signaling pathway (e.g., vesicular proteins, calcium channels) may be changed warrants further investigation.

These data suggest that the receptor exerts a tonic modulation of basal synaptic transmission by constantly and specifically shaping low frequency excitatory and inhibitory postsynaptic responses. This novel mode of action of the receptor takes place in near-physiological conditions, in addition to its classical low-pass filter action during enhanced synaptic activity. The receptor modulates both GABAergic and glutamatergic transmission in the thalamus, and its inhibition has consequences on animal behavior.

### ADX71743 Reveals the Potential Constitutive Activity of the mGlu7 Receptor

Our work indicates that endogenous WT mGlu7 receptors are tonically active. Due to the limitations brought by the available pharmacology we cannot exclude a contribution of elicited glutamate release to the activity of the receptor. However, we may deduce that at least part of the effect was due to an agonist-independent activity. Several arguments favor the existence of a constitutive activity of mGlu7. First, our results in HEK cells show that, in an isolated system where the receptor expression and glutamate concentration are tightly controlled, mGlu7 receptors can act in an agonist-independent manner. Furthermore, the glutamate concentration in GABA synapses is bound to be well below the EC50 of mGlu7 receptors (500 μM–1 mM, Schoepp et al., 1999) and yet mGlu7 responded to the application of the NAM in the TRN (phasic current) and VPM (tonic current). Interestingly, Turner and Salt (1999) showed that two orthosteric mGluR antagonists, MAP4 (acting on mGlu4, mGlu7, and mGlu8) and LY341495 (acting on mGlu2, mGlu3, mGlu4, mGlu7, and mGlu8), had no effect on evoked EPSP amplitude in thalamocortical slices, suggesting the absence of endogenous glutamate-dependent activity. We previously proposed the existence of a tonic mGlu7a receptor-mediated inhibition of voltage-gated calcium channels in cultured cerebellar granule cells, antagonized by binding of the protein kinase C substrate MacMARCKS to the receptor C-terminal tail (Bertaso et al., 2006). Recent data in dissociated superior cervical ganglia neurons showed that transfection of mGlu7a receptor is sufficient to induce spontaneous receptor intracellular signaling, and to inhibit voltage-dependent calcium channels (Kammermeier, 2015). However, the inverse agonist used in the study, MMPIP, seems to have a different effect depending on the cellular context (Niswender et al., 2010). The future discovery of a true, mGlu7-specific negative orthosteric antagonist will allow to confirm these results in a clear-cut way.

### Tonically Active mGlu7a Receptors as Gatekeepers against Aberrant TC Oscillations

What could be the physiological role of the mGlu7a receptor PDZ-dependent tonic activity? Genetic mouse models allowed a deeper understanding of the mGlu7 receptor synaptic functions. For example, the mGlu7 receptor KO and mGlu7a^AAA^ KI mice display different phenotypes (Sansig et al., 2001; Zhang et al., 2008), suggesting specific synaptic functions of mGlu7a receptor PDZ-dependent pathway. We found that in the TC network, mGlu7a receptor-expressing synapses display higher short-term synaptic depression than mGlu7a^AAA^ mutant synapses. Pelkey et al. (2008) showed that the combination of mGlu7a receptor activation and mossy fibers high frequency stimulation induces a PICK1-dependent long-term depression in hippocampus. It thus appears that the mGlu7a receptor-PDZ ligand signaling participates in both short-term and long-term plasticity, the neural basis of many cognitive and emotional processes (O’Connor et al., 2010). The PDZ interaction of mGlu7a with PICK1 is required for the receptor intracellular signaling, which could explain these effects, but whether or not control of the receptor tonic activity by PDZ interaction is required remains open.

**FIGURE 9:**
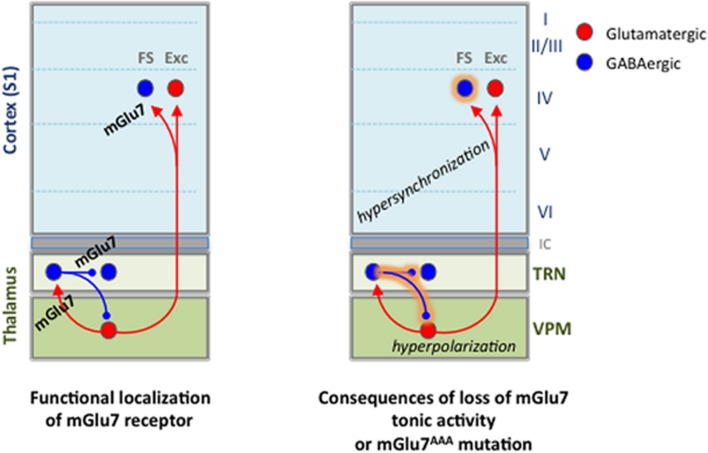
**Summary of findings on functional expression of mGlu7 receptors in the thalamocortical network and alterations brought by the loss of mGlu7 constitutive activity or by the mGlu7a^AAA^ mutation**.

Our data suggest an excessive activation of VPM → TRN, VPM → cortical layer 4 FS interneurons and intra-TRN synapses in mGlu7^AAA^ mice, with several consequences on thalamocortical network physiology. A tentative hypothesis is that such a tonic modulation of synaptic activities probably results from tonic activity of mGlu7 receptors. This may have the following consequences on the TC network functions (summarized in **Figure 9**):

(1) Over-activation of the TRN neurons by VPM should induce hyper-excitation of reticular neurons, and increase the inhibitory feedback of reticular neurons toward VPM nucleus via a recurrent pathway. This effect is amplified by the reticular neurons-mediated spontaneous phasic and tonic hyper-inhibition of thalamic cells. Interestingly, increased TRN neurons-mediated tonic inhibition has been observed in all the other absence epilepsy models (Cope and Di Giovanni, 2009). Hyperpolarization of VPM neurons is able to switch their firing pattern from tonic to burst mode, typically observed during absence seizures (Huguenard and McCormick, 2007). This would be more likely in mGlu7a^AAA^ as opposed to WT mice, where tonic activity of mGlu7 would provide a permanent control of those bursts.
(2) Intra-TRN inhibition is able to desynchronize reticular neurons activity (Beenhakker and Huguenard, 2009). Our results show that intra-TRN inhibition is increased in the young mGlu7a^AAA^ mice used in the present studied. This effect mediated by mGlu7a receptor PDZ interaction should therefore prevent synchronization of the TRN neurons and protect against absence seizures. Intra-TRN connections are known to slowly disappear during cerebral maturation in mice (Pinault, 2004). Therefore this protective effect would be lost in adult mice, which may support the difference in the age at which absence seizures appear in rodent models compared to human (Crunelli and Leresche, 2002).
(3) Layer 4 FS interneurons are known to mediate fast and powerful perisomatic IPSCs, and are responsible for the main feed-forward inhibition in the TC network (Porter et al., 2001; Cruikshank et al., 2007). Our experiments in mGlu7a^AAA^ TC slices suggest that mGlu7 receptor would prevent over-activation of VPM-FS interneuron synapses in cortical layer 4, and hyper-synchronization mediated by these interneurons (Swadlow et al., 1998).
(4) Several studies have highlighted the impact of tonic GABA_A_ current on the control of neuronal excitability, with implications in different pathologies such as epilepsies, stress or depression-related paradigms (Glykys and Mody, 2007). We demonstrate here a negative feedback provided by the mGlu7 receptor on tonic thalamic GABA_A_ current. Similarly, Errington et al. (2011) showed an inhibition of the tonic GABA_A_ current by mGlu receptors in thalamic neurons, but the concentrations of L-AP4 used in the study were too low to fully activate the mGlu7 receptor subtype. Given the low affinity of the receptor for glutamate, it is possible that in physiological conditions the mGlu7 receptor acts mainly via its tonic rather than phasic agonist-dependent activity, at least at GABAergic synapses where the concentration of glutamate is expected to be low.

All these data highlight the fact that the physiological function of the mGlu7 receptor seems of particular importance to modulate inputs onto GABAergic neurons. The phenotypic abnormalities found in mGlu7 receptor mutant models reflect the strength of these synaptic controls in WT CNS. It is also interesting to underline the emerging concept that mGlu7 is capable to inhibit synaptic transmission in a target-specific manner, as shown by the differential effect on synapses harboring from VPM neurons to either the cortical layer 4 excitatory neurons (no effect of mGlu7) or FS interneurons (inhibition). Interestingly, synapse-specific expression of mGlu7 has been found in hippocampal CA1 synapses onto interneurons expressing a postsynaptic protein, Elfn1 (Tomioka et al., 2014). Recently, Klar et al. (2015) have found that mGlu7 receptors control the feed-forward inhibition onto CA1 pyramidal neurons, acting on GABAergic parvalbumin-expressing presynapses. Incidentally, the lack of mGlu7-dependent inhibition observed in the VPM is in agreement with previous immunohistochemical data showing the absence of mGlu7 receptor in mouse VPM (Kinoshita et al., 1998).

An interesting question is raised by the consistently smaller response in some mGlu7a^AAA^ synapses compared to WT. These synapses displayed high EPSC or IPSC amplitudes (>50 pA) in WT mice, while no difference was observed in intra-TRN low amplitude postsynaptic currents. This could reflect a variety of mechanisms. First, the lack of mGlu7 receptor-mediated voltage-dependent Ca^2+^ channels inhibition in mutant mice might induce a sustained glutamate and GABA release by VPM and TRN axon terminals, respectively. Indeed, we showed that spontaneous GABA release by TRN neurons is increased in mutant mice. As TRN and cortical L4 neurons receive other glutamatergic inputs in addition to VPM afferents, we could not examine the VPM neuron spontaneous activity. Ca^2+^ accumulation into mGlu7 receptor-expressing axon terminals may also induce higher facilitation and decreased depression, as observed in mutant mice synapses. These effects might deplete the releasable pool of neurotransmitter in presynaptic sites, leading to a synaptic depression. Alternative explanations could be the internalization of postsynaptic receptors after sustained neurotransmitter release or a decrease in thalamic fiber excitability.

### mGlu7 Receptors at the Borders of Sleep and Epilepsy

A few studies have highlighted the link of mGlu receptors with absence seizures (Snead and Banerjee, 2000; Bertaso et al., 2008). *In vivo* administration of ADX71743 enhanced thalamic oscillations in association with drowsiness and behavioral arrest in WT mice. *In vitro* activation of mGlu7 by L-AP4 in rats reduces spindle oscillations, which typically occur during the early stage of sleep or during active phases of slow-wave-sleep oscillations (Kyuyoung and Huguenard, 2014).

As both sleep and absence seizures are supported by reciprocal connections between glutamatergic VPM and GABAergic TRN neurons, and somatosensory cortex (Steriade, 2006; Huguenard and McCormick, 2007), our data suggest that tonic activity of the mGlu7 receptor prevents unbalanced activity of the network that would lead to abnormal sleep-related behavior or seizures. The border between the lethargic effect and absence seizures seems blurry, as in a few animals the EEG revealed SWDs typical of epileptic seizures. The lack of effect of ADX71743 in mGlu7a^AAA^ mice suggests the PDZ ligand-dependent tonic activity as essential for receptor functions. Alternatively, one could imagine an occlusion mechanism between the mutation of the receptor PDZ ligand motif and the action of ADX71743.

Overall, it appears that the mGlu7 receptor modulates the global thalamic state of excitability, reducing its propensity to switch from the tonic to the oscillatory mode.

In human, *GRM7* gene polymorphisms have recently been associated with autism (Yang and Pan, 2013), schizophrenia (Ohtsuki et al., 2008), attention deficits and hyperactivity (Park et al., 2013), age-dependent auditive deficits (Friedman et al., 2009), although not yet to epilepsies (Goodwin et al., 2000). By acting directly at crucial nodes of oscillatory networks, the mGlu7 receptor could provide a new therapeutic target against epilepsies, and more generally neuropathologies resulting from unbalanced excitation/inhibition inputs.

## Author Contributions

VT, JP, LF, and FB designed the study and wrote the manuscript; VT, FB, and BG performed the electrophysiology, optogenetics, and pharmacology experiments; AC and BG performed the *in vivo* EEG recordings; PF designed the EEG analysis routine; DR and FA designed the synthesis and synthesized the ADX71743.

## Conflict of Interest Statement

The authors declare that the research was conducted in the absence of any commercial or financial relationships that could be construed as a potential conflict of interest.
