# Supplementary figures and images for: Phasic and Tonic mGlu7 Receptor Activity Modulates the Thalamocortical Network
Source: Front Neural Circuits. 2016 Apr 25;10:31. doi: 10.3389/fncir.2016.00031 (PMC4842779; doi:10.3389/fncir.2016.00031)

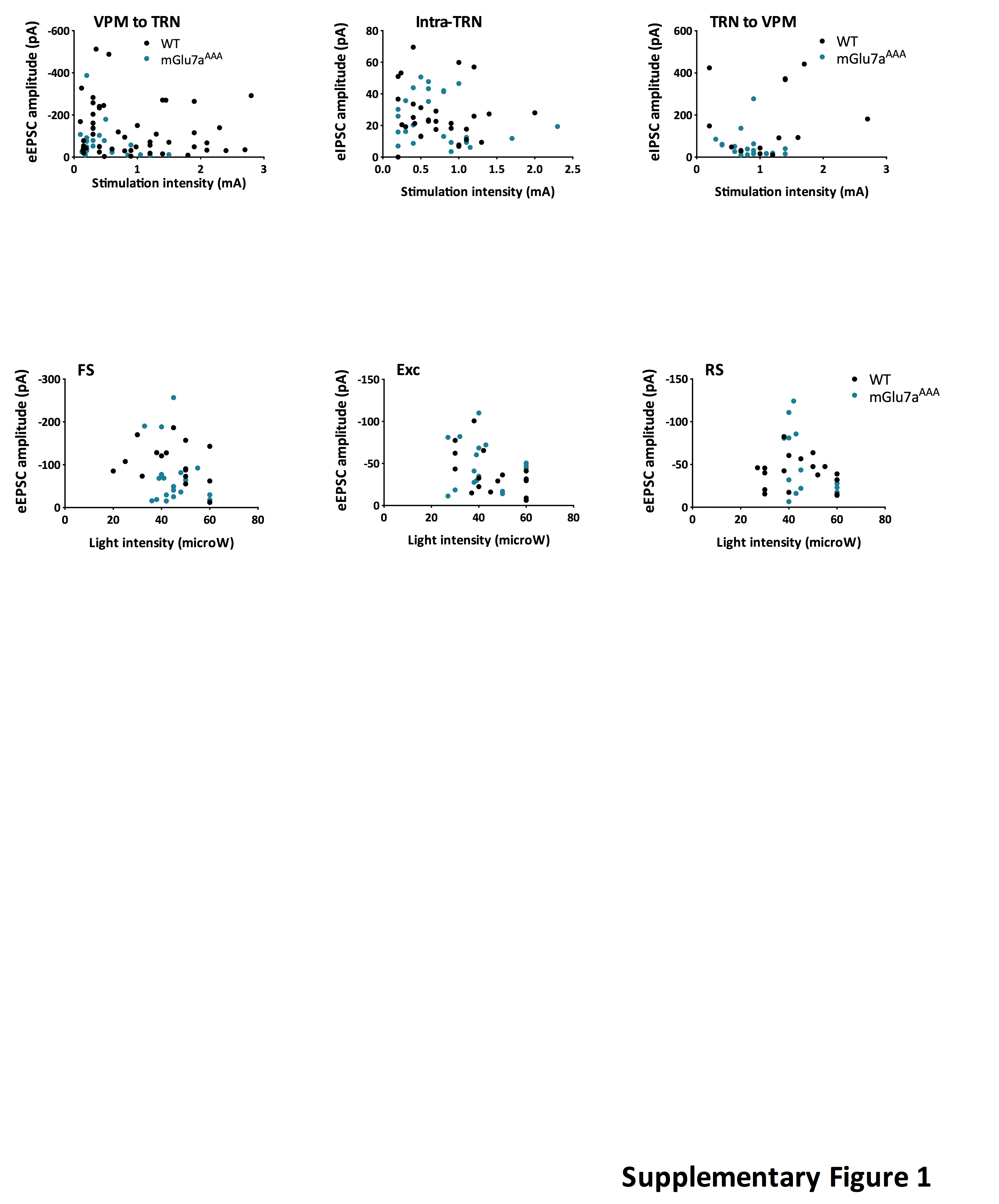

Supplement: Supplementary file 1 [file Image_1.TIFF]

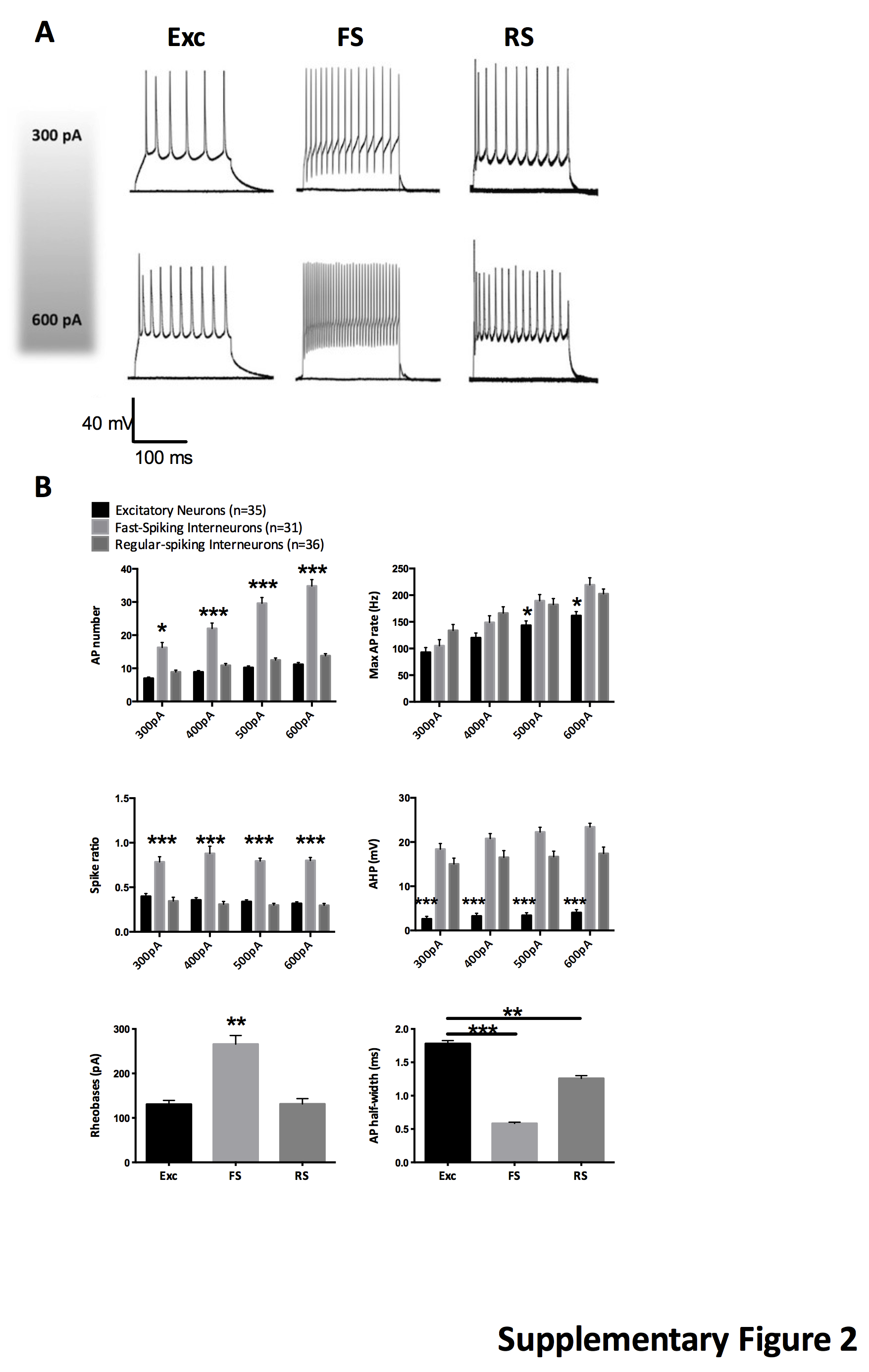

Supplement: Supplementary file 2 [file Image_2.TIFF]

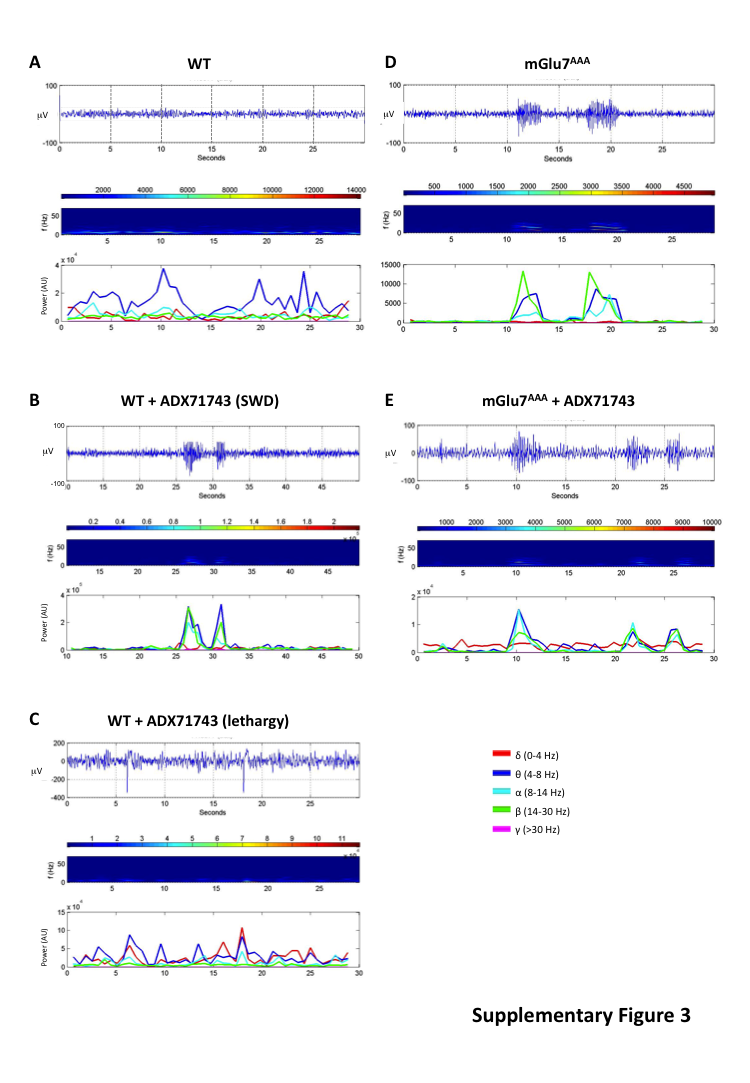

Supplement: Supplementary file 3 [file Image_3.TIF]
