# Supplementary material for: Phasic and Tonic mGlu7 Receptor Activity Modulates the Thalamocortical Network
Source: Front Neural Circuits. 2016 Apr 25;10:31. doi: 10.3389/fncir.2016.00031 (PMC4842779; doi:10.3389/fncir.2016.00031)
Supplement: Supplementary file 4 [file Image_4.PDF]

SUPPLEMENTARY FIGURE LEGENDS

Supplementary Figure 1. Graphs of stimulation intensity vs. eEPSC or eIPCS amplitudes are presented for the synapses indicated on top of each graph, for either WT or mGlu7a^{AAA} mice. VPM and TRN synaptic responses were obtained by electrical stimulation, FS, Exc and RS synaptic responses were obtained by blue light stimulation (488 nm) of hChannelrhodopsin.

Supplementary Figure 2. Electrophysiological properties of L4 neurons: classification in three classes.
(**A**) Representative voltage traces from cortical layer 4 excitatory neurons (i.e. pyramidal and spiny stellate cells, left), Fast-Spiking (middle) and Regular-Spiking (right) GABAergic interneurons responding to 300 pA (top) and 600 pA (bottom) injection. The different firing patterns reflect intrinsic properties of each neuron type. They are identical in WT and mGluR7^{AAA} mice (data not shown). (**B**) Histograms recapitulating the parameters used to discriminate between the neuron subtypes. Data from WT and mGluR7^{AAA} mice are pooled, and each graph show neuron firing properties to 300, 400, 500 and 600 pA current injection. Fast-Spiking interneurons firing is very different from excitatory and Regular-Spiking neurons: they display high-frequency action potentials (AP) with elevated after-hyperpolarisation potential (AHP) and low AP half-width. The spike ratio, which reflects the difference in inter-event interval between the two first and the four last AP, is high, illustrating that Fast-Spiking interneurons AP are non-adaptative. Their rheobase, which corresponds to the lowest current needed to induce at least one AP, is high, reflecting the fact that Fast-Spiking interneurons respond to elevated current stimuli only. On the other hand, Regular-Spiking interneurons display higher AP frequency and AHP than excitatory neurons, but lower AP half-width. Both excitatory and Regular-Spiking neurons show a low rheobase and a strong AP adaptation. * $P<0.05$, ** $P<0.01$, *** $P<0.001$, Mann-and-Whitney test. Error bars indicate SEM.

Supplementary Figure 3. (**A**-**E**) Example of EEG traces and corresponding short-time Fourier transform representation (frequency over time) and power of each frequency band as depicted in the bottom left corner, where where δ=2-4 Hz, θ=4-8 Hz, α=8-14 Hz, β=14-30 Hz, and γ=30-70 Hz. The different traces are examples for WT mice injected with either the vehicle alone (**A**) or the mGlu7 NAM (**B**, **C**) and for representative mGlu7^{AAA} mice showing spontaneous SWD when treated with the vehicle (**D**) or with the mGlu7 NAM. Traces in (**B**, **C**) show respectively SWD-like and low frequency activity with recurrent spikes corresponding to K-complexes. The level of wavelet power is represented using the color scale.
